# Use of email for patient communication in student health care: a cross-sectional study

**DOI:** 10.1186/1472-6947-5-2

**Published:** 2005-01-27

**Authors:** Johanna Castrén, Marja Niemi, Irma Virjo

**Affiliations:** 1 Medical School, Department of General Practice, 33014 University of Tampere, Finland; 2 Finnish Student Health Service, Töölönkatu 37, 00260 Helsinki, Finland; 3 Pirkanmaa Hospital District, Department of General Practice, 33521 Tampere, Finland; 4 STAKES, National Research and Development Centre for Welfare and Health, Box 220, 00531 Helsinki, Finland

## Abstract

**Background:**

Citizens increasingly use email in personal communication. It is not however clear to what extent physicians utilize it for patient communication. Our study was designed to examine physicians' activity in using email and to estimate the proportion of email messages missing from documentation in electronic patient records (EPR).

**Methods:**

All physicians (n = 76; 48 general practitioners and 28 specialists) at the Finnish Student Health Service received a questionnaire by email, and were asked to print it and keep a daily tally of visits, phone calls and email messages over the study period of one working week (5.5. – 9.5.2003). The response rate was 70%. The data originating from the questionnaire were compared with statistical data from the EPR during the study period.

**Results:**

The majority (79%, 41/52) of doctors reported using email with patients, averaging 8.6 (range: 0–96) email contacts and a percentage rate of "email / visit" 20% (range: 0–185%) in one working week. Doctors in the capital city region and those doctors who had a positive attitude toward email for patient communication were most active in email use. Up to 73% of email contacts were not documented in the EPR.

**Conclusion:**

The activity in using email with patients verified among Finnish physicians is compatible with recent study results elsewhere. The notable proportion of un-recorded email messages establishes the need for an electric communication system built into the EPR to improve the quality of patient care and to limit medico-legal risks.

## Background

Citizens increasingly use email in personal communication [1]. It is not however clear to what extent physicians utilize it for patient communication. In reports from the 1990s 1–14% of doctors in the USA and Norway used email in patient work [2,3]. In recent studies up to 73% of physicians had used email for patient communication [4-6].

International and national recommendations and guidelines have been published on email use between doctor and patient; the contents of the Finnish guidelines are well in line with European guidelines [7,8]. These guidelines emphasize the suitability of email for only certain limited purposes and stress the risks to information security.

Use of email between doctor and patient has been studied in one controlled and randomized and three cross-sectional studies [4,5,9,10]. In a study by Katz and colleagues the number of email contacts of physicians in their study group (46 email messages/100 scheduled visits) was greater than that in the control group (9/100) [5]. Houston and associates found that the majority of doctors received daily 1–5 email messages from their patients [9]. According to Sittig, physicians received daily in average 2.6 messages, and monthly an average of 40 per 140 visits [10]. Gaster and colleagues noted that physicians on average received 7.7 email messages in a month from their patients. Physicians in university clinics were most active in email use, while those in municipal primary health care were least active. Of physicians 58% reported in the questionnaire that the email contacts with patients were for the most part not registered in patient records [4].

Among Finnish citizens of working age young adults are the most active users of email and Internet [1,11]. University students use these electronic net services even more actively than the young adult population as a whole. In a study from 2002 99% of students reported using email and Internet at least weekly [12]. All students have an email address at the university and their health providers at the FSHS can be reached by email. The student health care system can be seen as an appropriate setting to use email for patient communication [13]. The students represent a young, well educated, relatively healthy part of population which has been identified to be the most active to use email in patient-doctor communication [2,5,14].

### The Finnish Student Health Service

The Finnish Student Health Service (FSHS) provides primary health care services to approximately 140.000 university students in Finland. The FSHS has health stations in 16 university cities. Services include health promotion, consultations with general practitioners and with other clinical specialists, mental health care, and dental care. Since 1993 FSHS has provided health counseling in Internet. Since 1999 all physicians have had an email account at their disposal in health stations and an email address of type: firstname.surname@yths.fi. Principles of communication by email with FSHS' employees and of other forms of electronic services (email service for cancellation of appointments, health counseling service on the Internet, and email service for feedback) are available at the FSHS' website.

The Social Insurance Institution, the university cities, the State of Finland, and the students themselves finance FSHS services. Students pay an annual obligatory health care fee as a part of the Student Union's membership fee. There is no other fee for preventive services, visits to general practitioner or public health nurse, and laboratory or X-ray examinations prescribed during these visits. Use of Internet services is also free of charge. The FSHS employs 560 persons and 63% of the physicians are general practitioners. In this paper general practitioners also include specialists of general practice/family medicine, whereas "specialists" refers to clinical specialists other than psychiatrists or oral surgeons.

### Aims of the study

The aim of the study was to seek answers to the following questions:

1. How actively did physicians at student health care use email in communication with their patients?

2. How much did they use email compared to phone calls and patient visits?

3. Who were the active doctors using email with patients?

4. What proportion of visits and phone calls could be candidates for substitution by email communication?

5. Did the volume of visits, phone calls and email messages documented in the EPR of the FSHS during the study period differ from that of visits, phone calls and email messages registered in the study?

## Methods

All physicians (n = 82) in the FSHS' functionary register in April 2003 received a questionnaire by email. We excluded six physicians, who were not any more working for the FSHS and took exception to the two authors. The actual number of survey population was 74. The questionnaire (see Additional file 1) included background factors and a registration (in form of daily tally) of numbers of patient contacts, phone calls and email messages over one working week. Respondents were also asked to assess the number of visits and calls replaceable by email, and the number of email messages including a request, which could not be fulfilled without face-to-face contact. Also doctors' attitudes toward email use for patient communication were asked.

The first mailing of questionnaire took place 28.4.2003 and a reminder was sent 5.5.2003. Recipients were asked to print the survey form, fill it in by hand, and return it by internal mail. Overall 52 out of 74 (70%) physicians returned a completed survey.

Respondents were grouped according to age, location, speciality licence, and type of employment (Table 1). Facts on years of birth were collected from the register book "Finland's Doctors 2002" and other background factors from the FSHS' functionary register.

**Table 1:** Background variables of all physicians at the FSHS and of the respondents.

	All physicians	Respondents
	(n = 76)	(n = 52)

	%	%

Gender		
Male	34	29
Female	66	71
Age (years)		
45 or under	33	31
46–55	39	42
56 or over	28	27
Location		
Capital city region	38	42
Turku and Tampere	28	27
Other^1)^	34	31
Speciality licence		
General practitioner	63	69
Specialist	37	31
Type of employment		
Permanent	68	85
Non-permanent^2)^	32	15

Distribution (%) of background variables of all physicians at the Finnish Student Health Service and of the respondents of the survey.

^1)^Small university towns

^2)^Vicars and fee-based working doctors

All physicians at the FSHS utilize EPR (Medicus^®^). We collected the numbers of patient contacts documented at Medicus^® ^for the study period using the statistical software Cognos^®^.

We entered data using Microsoft Excel^® ^software and performed statistical analysis using StatsDirect Statistical^® ^(3,2,7 -version) software. Statistical analyses were conducted using the proportion test for two independent groups, the χ^2 ^test, Fisher's exact test, the unpaired t-test, the Mann-Whitney test and the Kruskal-Wallis test. All tests were made two-sided and p-values below .05 were regarded as statistically significant.

## Results

### Respondents

Of all respondents 29% were men and 71% women (Table 1). The respondents and all doctors at the FSHS were compared according to the background variables. The doctors who answered represented well the overall body of physicians working in the FSHS. The mean age of male respondents was 52.8 (range 34–65) and of female 48.5 years (range 29–65). This was in the same range as the mean age of all doctors at the FSHS (men 51.3 and women 48.3 years).

### Activity of using email

In one working week 79% of doctors had used email and 98% the phone for patient communication. Respondents reported 2296 patient visits, 948 phone calls and 449 email contacts. They had on average 8.6 email contacts and 18.2 phone calls per week with their patients (Table 2). They reported a mean percentage for "email per visit" of 20%, and phone calls in proportion to visits ("phone call per visit") averaging 40%. Eleven doctors (21%) reported more email contacts than phone calls.

**Table 2:** Characteristics of the use of email.

Characteristic	Mean	Median	Minimum	Maximum
	n^1)^	n^1)^	n^1)^	n^1)^

Type of patient contact				
Visit	44.2	46.5	7	78
Phone call	18.2	18.0	0	59
Email	8.6	3.5	0	96
				
	%	%	%	%

Proportion of the respective contact types				
Phone call / visit	40	36	0	100
Email / visit	20	12	0	185
Email / phone call	55	22	0	600

Characteristics of the use of email in one week (5.5. – 9.5.2003) among physicians (n = 52) at the Finnish Student Health Service.

^1)^ Number of contacts

We tested the variables showing email usage and phone calls with respect to background factors. There were no statistically significant differences by gender, age group, speciality licence or type of employment. Of physicians in the capital city area 41% reported more email contacts than phone calls. Among doctors working in Turku and Tampere the proportion was 7.1%, and among those working in small towns 6.3%. The difference between capital city region and other locations was statistically significant (p = .015).

Respondents' general attitude toward email for patient communication was evaluated by the statement "Email contacts with patients facilitate my work." The group of "positives" was formed of the 56% of respondents who on a five step Likert scale replied: "same opinion" or "nearly same opinion."

The "positives" reported more email contacts than others (email per visit: median18% versus median 3%, p < .001). All eleven who had reported more email contacts than phone calls belonged to the "positive" group.

### Patient visits and phone calls replaceable by email

Doctors estimated that 2% (57/2296) of patient visits could have been replaced by email. Of phone calls 21% (196/948) could have been substituted with email. Respondents estimated that 10% (45/449) of patients' email messages required a personal consultation.

### Documentation in the EPR

The number of visits registered in the survey did not differ from the presumed number documented in the EPR (Table 3.). The difference noted between registered and presumed email contacts shows that 73% of email contacts were not entered in the EPR.

**Table 3:** Patient contacts documented in the EPR and registered in the survey.

Type of patient contact	Number of patient contacts			Statistical significance of the difference between expected and registered contacts^2^)
	
	All physicians	Respondents		
	
	Documented data in electronic patient record (Medicus^R^)	Expected^1)^	Registered	
	n	n	n	p

Visit	3098	2107	2296	0.237
Phone call	1115	758	948	< 0.001
Email	178	121	449	< 0.001

Patient contacts documented by all physicians at the Finnish Student Health Service (n = 76) in the electronic patient record (Medicus^R^) and registered by respondents (n = 52) in a survey over one working week (5.5. – 9.5.2003), as well as the statistical significance of the difference in contact numbers.

^1)^Based on the proportion of respondents among all physicians

^2) ^Proportion test for two independent groups

## Discussion

Even if university students do not represent the whole population, they can act as "pilot population" representing adults of working age of a future information society. Our study population – doctors taking care of students – was small with only 52 respondents. Thus the results of the study cannot be indiscriminately generalized. Because of the small study population comparison of the subgroups may not be reliable.

Although the study group was small, it well represented all the doctors at the FSHS, and the response rate was high. A further strength was that we compared the number of patient contacts documented in the questionnaires during the study week to the statistical data of contact numbers from the EPR at the same time. In other studies no corresponding comparison has been made.

The doctors were asked to keep a daily tally of visits, phone calls and email messages, and to evaluate how many visits or phone calls could have been replaced by email. Many doctors undoubtedly did this simultaneously with patient work. Some doctors might have been in a hurry, they probably supplemented the questionnaire at the end of the day. To achieve a more accurate evaluation of visits and phone calls replaceable by email, a continuous assessment (visit by visit, phone call by phone call) could have been stressed even more in the instructions.

The doctors at the FSHS do not have a specific electronic communication system focused on patient communication. They use their general, unprotected email system also to communicate with patients. A specific communication system used only for patient communication would enable an automatic collection of the patient communication data and create a more accurate database than our data collection method.

Katz and colleagues have made the only controlled and randomized study concerning physicians' use of email [5]. Our own results on the average number of doctors' email contacts and email usage are of the same magnitude as those referred to above and in other recent studies in the USA [4,9,10]. In the present study 79% of respondents used email for patient communication. The proportion of those who had used email is clearly larger than in older studies, and at the same level as reported in recent international studies [2-6].

Our study revealed individual differences in the use of email in patient work. Differences in physicians' activity in using email have previously been reported in only one study [4]. Deriving of our small study group only especially glaring association between subgroups of respondents could be verified. Our findings still support the results published by Gaster and associates. Physicians working in the capital area were more active email users than their colleagues elsewhere in Finland.

Physicians reckoned that email could replace only 2% of visits. This confirms Sittig's evaluation in 2003 that email could possibly cover a small percentage of visits [10]. Increasing the use of email can thus not considerably reduce the number of patient visits. On the other hand it could make physicians' crowded telephone hours easier [15].

When we compared contacts in the EPR with contacts registered daily on the questionnaires we found that the majority of email contacts were not registered in the EPR. This finding is supported by Gaster and colleagues who asked physicians themselves to describe how often they usually registered email contacts in patient records [4].

FSHS provides specific electronic services for focused issues (email service for cancellation of appointments, health counseling service on the Internet, and email service for feedback). Principles of recommended issues to use email between health providers and patients are available for students at FSHS' websites. We have had a presumption that email messages between FSHS' physicians and their patients mainly handle patient care. Nyström's congress report from 2004 supports our presumption. He explored 139 email messages from 103 individual patients at his GP practice at the FSHS and noticed that 77 % of email messages handled medical tests, and 16 % handled follow-ups of symptoms or illnesses [16]. Thus the information in email communication should be entered in patient records.

All university students in Finland have access to Internet and email at their universities. Use of email as communication method in health care does not in their case cause inequalities in health. A general tendency in the societies to provide also health services widely in electronic form (in Internet or by email) can contribute to inequalities for those who are not able to use modern technologies [13,17].

## Conclusions

Doctors at the FSHS had an average of 8.6 email contacts with their patients during one week. The proportion of email contacts per visit was 20%. Physicians estimated that email contacts could substitute 2% of patients' visits and 21% of phone calls. Of email contacts 73% were not documented in the EPR.

Our study indicates that email communication really constitutes a part of patient work. This should be taken into account in planning working time and daily timetables. Use of software not integrated with the EPR increases the physician's registering load and currently involves extra work. It is not possible to confirm the patient's identity reliably using two separate systems. A system allowing retrieval of patient's identity safely and with no need to register separately the email communication in the EPR would promote the patient's adequate treatment and reduce the physician's medico-legal risks.

There is a need for a larger study on email utilization between patient and physician which better covers the medical profession. The consumer point of view should also be better taken into account. A content analysis of email messages for patient communication combined with assessments of email documentation in the EPR could have strengthened present study regarding the importance of email registration in patient records.

In Finland the Ministry of Social Affairs and Health has instigated a major project to safeguard health care [18]. One part of this programme demands that the whole public health care field should be using EPR by 2007 [19]. More research data are needed on electronic communication with patients and on users' experiences. The future EPRs should include a purposeful, safe and secure means of patient communication.

## Competing interests

The author(s) declare that they have no competing interests.

## Authors' contributions

JC participated in the design of the study, coordinated it, keyed data into computer, performed the statistical analysis and drafted the manuscript with co-authors. MN conceived of the study, participated in the design of the study and wrote parts of manuscript in English. IV participated in the design of study and drafted the manuscript. All authors read and approved the final manuscript.

## Pre-publication history

The pre-publication history for this paper can be accessed here:



## Supplementary Material

Additional File 1 Questionnaire Click here for file
