# Supplementary material for: Use of email for patient communication in student health care: a cross-sectional study
Source: BMC Med Inform Decis Mak. 2005 Jan 27;5:2. doi: 10.1186/1472-6947-5-2 (PMC548681; doi:10.1186/1472-6947-5-2)
Supplement: Additional File 1 — Questionnaire [file 1472-6947-5-2-S1.doc]

Name of respondent: (NOT disclosed to the researcher of data on sections A, B and C!)

A. Please answer the following questions by circling the correct alternative or by filling in the blanks:

| *Sex* | *Year of birth* | *Health centre* | *Position* | *Type of employment* |
| --- | --- | --- | --- | --- |
| 1 female  2 male | 19 |  | 1 GP  2 specialist | 1 permanent 2 temporary  3 fee-based |

B. Please tick the closest alternative out of the following:

| *Statement* | *Fully agree* | *Partly agree* | *Partly disagree* | *Fully disagree* | *Don't know* |
| --- | --- | --- | --- | --- | --- |
| 1. Email contacts with patients facilitate my work. |  |  |  |  |  |
| 2. E-mail security is good enough to guarantee reliable communication with patient. |  |  |  |  |  |
| 3. When communicating test results (e.g. lab), e-mail (sufficiently secure) should be favoured. |  |  |  |  |  |
| 4. Should a doctor wish to attend to patients by e-mail, it must be separately agreed upon by both doctor and patient. |  |  |  |  |  |
| 5. Should a patient wish to be attended to by e-mail, it must be separately agreed upon by both doctor and patient. |  |  |  |  |  |
| 6. Doctors need to be provided with written guidelines, general principles, on the use of e-mail in patient care. |  |  |  |  |  |
| 7. Patients need to be provided with written guidelines, general principles, on the use of e-mail in patient care. |  |  |  |  |  |
| 8. Use of e-mail in patient care must always be preceded by personal contact at the consultation hour. |  |  |  |  |  |
| 9. My work schedule allows sufficient time for e-mail contact with patients. |  |  |  |  |  |

C. List five uses for which you think e-mail (sufficiently secure; cf. Internet banking) is / might be ideal in patient care at FSHS.

| 1 | 2 | 3 | 4 | 5 |
| --- | --- | --- | --- | --- |

D Indicate (using numbers or other simple means) in the table the following (for week 19; 5–9 May 2003):

|  | *Mon 5 May 2003* | *Tue 6 May 2003* | *Wed 7 May 2003* | *Thu 8 May 2003* | *Fri 9 May 2003* |
| --- | --- | --- | --- | --- | --- |
| *Number of patient visits* |  |  |  |  |  |
| *Number of phone calls to/from patients* |  |  |  |  |  |
| *Number of e-mail contacts with patients* |  |  |  |  |  |
| *Of the above, patients called in because handling by e-mail WAS IMPOSSIBLE* |  |  |  |  |  |
| *Estimate how many patientvisits could have been attended to by e-mail* |  |  |  |  |  |
| *Estimate how many phone calls could have been replaced by e-mail contact* |  |  |  |  |  |

MANY THANKS FOR YOUR CO-OPERATION!
